# Ethno-medicinal documentation of polyherbal medicines used for the treatment of tuberculosis in Amathole District Municipality of the Eastern Cape Province, South Africa

**DOI:** 10.1080/13880209.2016.1266670

**Published:** 2017-01-31

**Authors:** Elizabeth Bosede Famewo, Anna Maria Clarke, Anthony Jide Afolayan

**Affiliations:** Faculty of Science and Agriculture, University of Fort Hare, Alice, South Africa

**Keywords:** Apiaceae; *Mycobacterium tuberculosis*, traditional healers

## Abstract

**Context:** Tuberculosis (TB) has remained a devastating global public health problem. In the continuing search for effective treatment, polyherbal remedies used as alternative medicines in the Eastern Cape Province of South Africa were surveyed.

**Objective:** The survey collected information and documents the list of ingredients such as the name of the plants used including the non-herbal inclusions, type and dosage of polyherbal formulations used for the treatment of TB.

**Materials and methods:** The survey was conducted over a period of 6 months using semi-structured questionnaires amidst informal conversations with the traditional healers in five communities in the study area. The chosen study area is the third infected Province with TB in South Africa.

**Results:** A total of nine polyherbal preparations were collected. Information on the parts of the plant used, mode of preparation and the dosage used were documented. In total, the herbs belong to 20 families of which Apiaceae, Liliaceae, Strychnaceae, Rutaceae and Hypoxidaceae are the most prominent. However, members of Apiaceae were commonly mentioned for the preparation of the remedies. The two most frequently used plants were *Allium sativum* L. (Liliaceae) and *Strychnos decussata* (Pappe) Gilg. (Strychnaceae). Rhizome was the commonest parts used, followed by the roots and barks.

**Conclusions:** This paper provides significant ethno-medicinal information on polyherbal medicines used for the treatment of TB in the study area. The therapeutic claims made on medicinal plants used for the preparations are well supported by the literature, with many of the species having antimicrobial properties.

## Introduction

Tuberculosis (TB) caused by *Mycobacterium tuberculosis* has remained one of the most prevalent causes of mortality in developing nations, especially in the Asian and African continents. The disease remains a big problem in these countries probably due to inadequate means for the management and treatment of the disease. According to World Health Organization (2002), about 95% of the approximately eight million cases of TB occur each year in Africa having the highest incidence. However, South Africa is the third highest incidence country after India and China with about 80% of the population infected with the disease. This rate has increased by 400% over the past 15 years most especially among the people living with human immuno-deficiency virus (HIV) (Lall & Meyer 1999; Green et al. 2010).

The conventional medical treatment for TB consists of a regimen of antibiotics taken over the course of several months. For this reason, many people especially those living in the rural communities do not strictly adhere to the plan for effective treatment; thus making the disease difficult to treat with conventional medicines (Lange et al. 2014). In addition, the side effects such as gastrointestinal upset, hepatitis, drug interactions and hearing loss associated with the consumption of these drugs have discouraged many people from continual use of the orthodox medicines (Laxminarayan et al. 2006). Also, the emergence of multi-drug resistant tuberculosis (MDRTB: resistant to the two most effective first-line drugs, isoniazid, and rifampin), extensively drug-resistant tuberculosis (XDRTB: MDRTB with additional resistance to at least one fluoroquinolone and one injectable drug) and totally drug-resistant (TDR) tuberculosis have made TB exacerbated, thus becoming a global health problem. The continual resistance of *M. tuberculosis* to commonly prescribed antimicrobials has made people fall back on herbal medicines for various therapeutic purposes (Orodho et al. 2011).

The inhabitants of the Eastern Cape Province have a long history of traditional plant usage for the treatment of various diseases including TB (Grierson & Afolayan 1999). In fact, about 30 plants belonging to 21 families are used by the traditional healers for the treatment of TB and associated diseases (Lawal et al. 2014). These plants are commonly combined with polyherbal remedies, which are prescribed in different formulations. However, the lists of ingredients used for the polyherbal formulations in the study area are yet to be documented. This information is very important in the development of serious armament for the treatment of TB, since a greater number of TB patients depend on traditional herbalists for their medical needs. Therefore, the aim of this study is to collect information and document the list, type, dosage and nature of the polyherbal medicines used for the treatment of TB in this Province, and to collect the information on the ingredients of the polyherbal formulations such as the name of the plants used including the non-herbal inclusions present in each remedy.

## Materials and methods

### Study area

**Figure 1.**
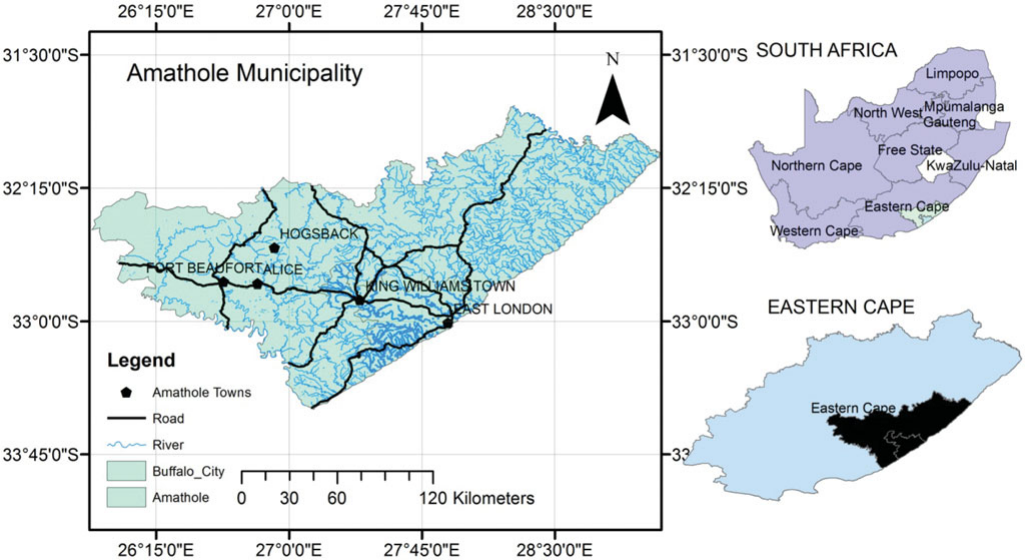
Map of Amathole District Municipality. (Source: Famewo et al. 2016)

The present study was carried out in five communities within the Amathole District Municipality of the Eastern Cape Province, South Africa (Figure 1). The area falls within latitudes 30°00′ to 34°15′S and longitudes 22°45′ to 30°15′E. It is bounded by the sea on the east and the drier Karroo (semi-desert vegetation) in the west. The elevation ranges from sea level to approximately 2200 m in the north of the province. The Amathole District Municipality lies at the heart of the Eastern Cape Province. The District stretches from the Indian Ocean coastline in the south to the Amathole Mountains in the north, and from Mbolompo Point (south of the Hole-in-the-Wall along the Transkei Wild Coast) in the east to the Great Fish River in the west. Presently, about 1.7 million people live in the study area (Afolayan 2003) including Africans (91%), coloreds (3%) and whites (6%). The main tribes of the area are Xhosa-speaking people(s) who are divided into several tribes with related but distinct heritages (Dyubeni & Buwa 2012).

### Data collection

The survey on the polyherbal medicines was conducted between March and August 2015 through semi-structured questionnaires amidst informal conversations with the traditional healers who use medicinal plants for the treatment of TB (Ajibesin et al. 2012; Asiimwe et al. 2013). The survey was carried out among the herb healers because they inherited the knowledge from their forefathers, therefore, passing it from one generation to another generation. The interviews were conducted in Xhosa, the local language of the informants, and English. The remedies were already prepared with water by the herbal healers into clean 2 L containers. They were purchased and the following information was collected for proper documentation; the local name of the herbs used for the polyherbal formulations, plant parts used, methods of preparation, modes of administration of the herbal remedy, doses and duration of treatment.

## Results and discussion

This study revealed that polyherbal remedies play an important role in healthcare delivery in South Africa, especially among the people living in the rural settings of the Eastern Cape Province. The application of these remedies in the management or treatment of diseases covers a wide range of conditions from chronic conditions, psychosocial problems, acute conditions, generalized pain, TB and HIV infection and acquired immune deficiency syndrome (AIDS). Ethnobotanical surveys have extensively studied South African populations of adults and children with several illnesses (Bodkin 2003).

A total of nine polyherbal medicines used for the treatment of TB were recorded and collected from the study area (Table 1); these are from East London (EL), King Williams Town (KWT) and Fort Beaufort (FB). Others are from Alice (AL) and Hogsback (HB). Each remedy was labelled and coded according to the place of collection; namely King Williams Town site A (KWTa), King Williams Town site B (KWTb), King Williams Town site C (KWTc), Hogsback first site (HBfs), Hogsback second site (HBss), Hogsback third site (HBts), East London (EL), Alice (AL) and Fort Beaufort (FB). The small number of remedies obtained in this study was because only a few traditional healers treat TB. They claim to have acquired the knowledge from their ancestors, and this knowledge has been transferred from one generation to another. The herbal healers in the Province diagnosed TB based on patients’ signs and symptoms before commencing with the treatment. Patients are carefully observed and symptoms such as blood in the sputum, prolonged coughing, chest pain and breath shortness including weight loss are taken as confirmation of TB.

**Table 1.** Polyherbal medicines used for the treatment of TB in Amathole District Municipality, Eastern Cape Province, South Africa.

Name code	Ingredients	Botanical name	Family	Parts used	Methods of preparation, administration and dosage
AL	Mountain garlic	*A. sativum* (L.)	Liliaceae	Rhizome	Infusion; Take 100 mL of the herbal mixture orally twice in a day for a period of 5–8 weeks.
	Mlomo mnandi	*Glycyrrhiza glabra* (L.)	**Fabaceae**	Root	
	Red carrot	*Daucus carota* (L.)	Apiaceae	Root	
	Inongwe	*Hypoxis argentea* (Fiscand)	Hypoxidaceae	corms	
	Mnonono	*S. decussate* (Pappe) Gilg	Strychnaceae	Bark	
	River pumpkin	*Gunnera perpensa* (L.)	Gunneraceae	Rhizome	
	Herbal menthol leaf	*Mentha piperita* (L.)	Lamiaceae	Leaf	
	Herbal buchu water	*Agathosma betulina* (Berg)	Rutaceae	Leaf	
EL	Inongwe	*Hypoxis argentea* (Fiscand)	Hypoxidaceae	corms	Infusion; Take 100 mL of the remedy orally thrice in a day for a period of 5–8 weeks.
	Intelezi	*Haworthia reinwardtii* (Haw)	Xanthorrhoeaceae	Leaf	
	Ngcambumvuthuza	*Ranunculus multifidus* (Forssk)	Ranunculaceae	Root	
	Inqwebeba	*Albuca flaccid* (Jacq.)	Asparagaceae	Leaf	
	Iqwili	*Alepidea amatymbica (*Eckl. & Zeyh.)	Apiaceae	Rhizome	
FB	Buchu leaf	*Agathosma betulina* (Berg)	Rutaceae	Leaf	Decoction; Take 75 mL of the remedy orally thrice in a day for a period of 5–8 weeks.
	Mountain garlic	*A. sativum* (L.)	Liliaceae	Rhizome	
	Ginger	*Zingiber officinalis* (L.)	**Zingerberaceae**	Rhizome	
	Chilli pepper	*Capsicum annuum* (L.)	Solanaceae	Vegetable	
KWTa	Maphipha	*Rapanea melanophloeos* (L.)	Primulaceae	Bark	Infusion; Take half a cup thrice in a day for a period of 5–8 weeks.
	Mnonono	*S. decussate* (Pappe) Gilg	Strychnaceae	Bark	
	Ixonya	*Kniphofia drepanophylla* (Baker)	Asphodelaceae	Root	
	Inongwe	*Hypoxis argentea* (Fiscand)	Hypoxidaceae	Corms	
	Sicimamlilo	*Pentanisia prunelloides* (Klotzsch)	Rubiaceae	Rhizome	
	Iphuzi	*Centella eriantha* (Rich.)	Apiaceae	Rhizome	
KWTb	Umdlavuza	*Lauridiatetragonia* (L.f.)	Celastaceae	Root	Infusion; Take 100 mL of the polyherbal remedy orally thrice in a day for a period of 5–8 weeks
	Mnonono	*S. decussate* (Pappe) Gilg	Strychnaceae	Bark	
	Inceba emhlophe	*Hermannia* sp. (L.)	Malvaceae	Root	
KWTc	Mnonono	*S. decussate* (Pappe) Gilg	Strychnaceae	Bark	A little quantity of the herb must be chewed immediately after the polyherbal remedy in KWT B has been administered, thrice in a day for a period of 5–8 weeks.
HBfs	Red carrot	*Daucus carota* (L.)	Apiaceae	Root	Infusion; Take 75 mL of the polyherbal remedy orally thrice in a day for a period of 3–5 weeks
	Mlungu mabele	*Zanthoxylum capense* (Thunb.)	Rutaceae	Bark	
	Calmoes	*Acorus calamus* (L.)	Acoraceae	Rhizome	
	Mountain garlic	*A. sativum* (L.)	Liliaceae	Rhizome	
HBss	Buchu leaf	*Agathosma betulina* (Berg)	Rutaceae	Leaf	Decoction; Take 75 mL of the herbal mixture orally thrice in a day for a period of 3–5 weeks
	Chilli pepper	*Capsicum annuum* (L.)	Solanaceae	Vegetable	
HBts	Maphipha	*Rapanea melanophloeos* (L.) Mez	Primulaceae	Bark	Infusion; Take 75 mL of the herbal mixture orally thrice in a day for a period of 3–5 weeks
	Red carrot	*Daucus carota* (L.)	Apiaceae	Root crop	
	Uroselina	*Cinnamomum camphora* (L.) J. Presl	Lauraceae	Bark	
	Mountain garlic	*A. sativum* (L.)	Liliaceae	Rhizome	

AL: Alice; EL: East London; FB: Fort Beaufort; KWTa: King Williams Town site A; KWTb: King Williams Town site B; KWTc: King Williams Town site C; HBfs: Hogsback first site; HBss: Hogsback second site; HBts: Hogsback third site.

The importance of Fabaceae lies in their effectiveness in the treatment of a wide variety of human ailments such as allergy, cough, hiccups, stomach ulcers, viral fevers, wounds and swellings (Padal et al. 2013). They also possess high level of biological activity due to the variety of chemically active constituents such as tannins, flavonoids, alkaloids, and terpenes present in members of this family (Molares & Ladio 2011).

Zingiberaceae is rich in substances having therapeutic value such as terpenoids, tannins and flavonoids. The rhizomes of this family are aromatic, tonic and stimulant. They are used as antimicrobial, antiarthritic, antioxidant, anticancer, antiinflammatory, antidiabetic, neuroprotective and larvicidal agents (Victório 2011).

**Figure 2.**
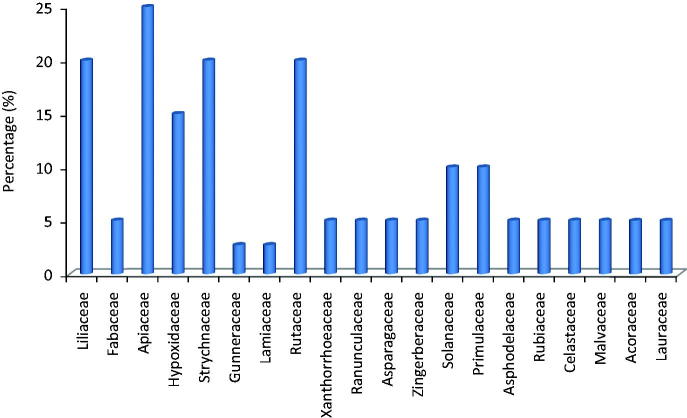
Frequency of the most used plant families in the preparation of polyherbal medicines for the treatment of TB in the study area.

The polyherbal remedies in the study area were prepared from 24 plants. The six most commonly mentioned plant families used in the preparation of the polyherbal formulations in terms of number and percentage are Apiaceae [5(25%)], followed by Liliaceae [4(20%)], Strychnaceae [4(20%)], Rutaceae [4(20%)], Solanaceae [2(10%)] and Primulaceae [2(10%)] (Figure 2). Members of Apiaceae have been previously cited as the most commonly used in this Province not only for the treatment of TB but also as an antidote for influenza, hypertension and to expel intestinal worms (Bisi-Johnson et al. 2010). The therapeutic claims made on most of the medicinal plants used for the preparation of these remedies are well supported by the literature, with many of the species having antimicrobial properties (Buwa & Afolayan 2009; Green et al. 2010).

**Figure 3.**
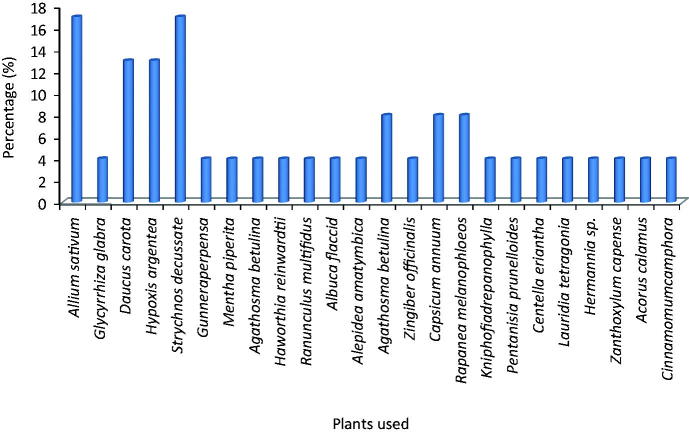
Occurrence of plant species used for the preparation of polyherbal medicines for the treatment of TB in the study area.

The most frequently used plant parts in this study was rhizomes [10(27%)], followed by bark [8(22%)], and roots [8(22%)]. Others are leaves [6(16%)], corms (3(8%)] and vegetable [2(5%)] as shown in Table 1. The four most frequently used plants are *Allium sativum* L. (Liliaceae) [4(17%)], *Strychnos decussata* (Pappe) Gilg. (Strychnaceae) [4(17%)], *Daucus carota* L. (Apiaceae) [3(13%)] and *Hypoxis argentea* (Fiscand) (Hypoxidaceae) [3(13%)]. Other plants such as *Agathosma betulina* (Berg) (Rutaceae) [2(8%)], *Capsicum annuum* L. (Solanaceae) [2(8%)] and *Rapanea melanophloeos* L. (Primulaceae) [2(8%)] are used for a few polyherbal preparations in the Province as represented in Figure 3.

The polyherbal medicines were prepared mainly by infusion [6(67%)] and decoction [2(22%)] with the exception of *S. decussate,* which should be chewed immediately after KWTb remedy has been administered (Table 1). This is an indication that the Eastern Cape traditional herbal healers use water mainly for the preparation of anti-TB treatments. These methods of extraction have been adopted from the ancient time. They seem to yield active principles required to treat TB (Nguta et al. 2015). The internal method of administration, which is by oral, was the main method of administrating all the remedies.

The diverse uses of each plant can be explicated by the fact that, a single plant can serve many medicinal purposes or perform different functions (Lawal et al. 2010). However, the combination of these herbs in polyherbal medicines probably results in better therapeutic activities and reduces the toxicity of such remedies. Naturally, polyherbal remedies contain multiple active constituents, which act synergistically against infections (Bhope et al. 2011). A similar study by Amodu et al. (2013) revealed that polyherbal medicines were effective against *Mycobacterium tuberculosis*. Probably, these therapies have gained popularity in both developed and developing countries because of their natural origin and fewer side effects for the treatment of various chronic and acute ailments (Ahmad et al. 2006; Benzie & Wachtel-Galor 2011). Polyherbal medicines are current pharmacological principle having the advantage of producing maximum therapeutic efficacy with minimum side effects to the consumers (Ebong et al. 2008). The cross-cultural acceptance and use of polyherbal remedies in different geographical zones is an indication of the potential of polyherbal as future sources of new classes of drugs against TB.

## Conclusions

This paper provides significant ethno-medicinal information on polyherbal medicines used for the treatment of TB in Amathole District Municipality of Eastern Cape Province. Africa is endowed with a biodiversity of medicinal plants, many of which are currently used in the traditional management of TB. The study shows that people in the study area still depend on polyherbal medicines for the treatment and management of TB. The documented remedies reflect rich ethno-medicinal knowledge in the province. However, further test are required to validate the ethno-medicinal usage of these polyherbal remedies as anti-TB agents.
